# Effects of Low-Intensity Pulsed Ultrasound on Knee Osteoarthritis: A Meta-Analysis of Randomized Clinical Trials

**DOI:** 10.1155/2018/7469197

**Published:** 2018-07-15

**Authors:** Xiao-Yi Zhou, Xiao-Xi Zhang, Guan-Yu Yu, Zi-Cheng Zhang, Fei Wang, Yi-Lin Yang, Ming Li, Xian-Zhao Wei

**Affiliations:** ^1^Department of Orthopaedics, Changhai Hospital, The Second Military Medical University, Shanghai 200433, China; ^2^Department of Neurosurgery, Changhai Hospital, The Second Military Medical University, Shanghai 200433, China; ^3^Department of Colorectal Surgery, Changhai Hospital, The Second Military Medical University, Shanghai 200433, China

## Abstract

**Background:**

Osteoarthritis (OA) is a common degeneration disease characterized with joint pain. The aim of the present study was to systemically review the effects of LIPUS on pain relief and functional recovery in patients with knee osteoarthritis (OA).

**Methods:**

PubMed, Embase, and Cochrane Library were searched manually for researches on LIPUS treatment in patients with knee OA from 1945 to July 2017. Two investigators independently selected the studies according to the inclusion and exclusion criteria, extracted the concerned data, and assessed the included studies. Meta-analysis was performed to evaluate VAS, WOMAC, and ambulation speed between control and LIPUS groups.

**Results:**

Five studies were selected in this study. Compared with control group, LIPUS group received a decrease of pain intensity with moderate heterogeneity (-0.79, 95% CI, -1.57 to 0.00; I^2^ = 65%, P = 0.04) by VAS and improvement in knee function by WOMAC (-5.30, 95% CI, -2.88 to -7.71; I^2^ = 44%, P = 0.17). No significant improvement was found in ambulation speed (0.08 m/s, 95% CI, -0.02 to 0.18 m/s; I^2^ = 68%, P = 0.03).

**Conclusion:**

The present study includes 5 high quality randomized controlled trials. The result indicated that LIPUS, used to treat knee OA without any adverse effect, had a beneficial effect on pain relief and knee functional recovery. More evidence is needed to prove whether LIPUS is effective in improving walking ability.

## 1. Introduction

Osteoarthritis (OA) is a common degeneration disease characterized with joint pain, stiffness, reduced range of motion, and swelling of the joint in adults globally [1, 2]. Chronic musculoskeletal diseases are one of the most common causes for OA [3]. The population suffered from OA increases with age. Researches revealed that more than 75% of the population over 65 years old are affected by OA to some extent. More than 186 billion dollars were the cost to treat patients with OA annually as estimated by the World Health Organization [4].

There remains a paucity of therapy to prevent OA from progression and damage the joint structure [5, 6]. Clinically, guidelines for the management of OA suggested conservative and pharmacologic treatments to relieve pain and prevent disease progression. Surgery is the final choice if the therapies above are not responded [7, 8]. However, disadvantages still exist for these therapies, such as the expensive cost, adverse effects of drugs, and invasive damage for patient's body. Therefore, a cost-effective and safe adjuvant method to treat OA is in urgent need.

Low-intensity pulsed ultrasound (LIPUS), a noninvasive therapy to treat fresh fracture and nonunion, has been approved by Food and Drug Administration in the United States for over 20 years. With the intensity < 100 mW/cm^2^, frequency of 1.5 MHz, and duty cycle of 20%, LIPUS shows no known adverse effects during treatment. Biological effects of ultrasound can be split into two parts: thermal effects and nonthermal effects. LIPUS would not cause tissue damage because its intensity is quite lower than usual. Various studies have investigated the effects of LIPUS on the treatment of OA [9]. The primary outcomes mainly focused on pain relief and functional recovery. However, it is still uncertain whether LIPUS has a beneficial effect in treating patients with knee OA.

Therefore, the present study was aimed to perform a meta-analysis on the efficacy of LIPUS in patients with knee OA through pooling high quality clinical trials.

## 2. Methods

### 2.1. Literature Search

Two reviews searched all the published randomized controlled trials with a combination of title words and abstracts words related to LIPUS and OA in the database of PubMed, Embase, and Cochrane Library. The search strategy was illustrated in the supplementary file (see the Appendix). The latest search was performed in July 2017 to determine the retrieved articles. The keywords for search strategy were adapted according to different database and Mesh.

### 2.2. Inclusion and Exclusion Criteria

Two reviewers independently identified the “Methods” section in all the eligible articles. The inclusion criteria are as follows: (1) randomized controlled trials; (2) patients with knee osteoarthritis; (3) studies containing at least one group using LIPUS as an intervention; (4) outcomes related to pain and function of patients; and (5) English literature. The exclusion criteria are as follows: (1) animal studies; (2) abstract, letter, review, systemic review/meta-analysis, or case report; and (3) non-English literature.

Both the reviewers reached an agreement on the selected articles for further analysis in the present study.

### 2.3. Quality Assessment

The included articles were independently and critically reviewed and evaluated by two reviewers with disagreement settled by a senior author. Quality assessment of included articles was based on Jadad method [10], which is a five-point questionnaire that scores randomized clinical trials on a 0-to-5 scale based on a number of factors such as randomization, blinding of the subject, and withdrawals or dropouts. Higher score indicates higher quality and evidence level of the clinical trial in the article.

### 2.4. Data Extraction and Outcome Measures

The objective of the present study was to identify the effectiveness of LIPUS on treating knee OA. Thus, the WOMAC scale is preferred to assess the improvement of knee function. In addition, ambulation speed (AS) is another important index to evaluate the functional improvement in patients. The changes related to pain management after LIPUS/sham LIPUS treatment are assessed by VAS score.

The reviewers extracted all relevant data separately. The extracted and tabulated data included first author, year of publication, nationality, patient demographics, duration of LIPUS application, Kellgren-Lawrence Grade, VAS score, Lequesne's index, and WOMAC. All extracted data were collected into a specific data form according to the study by Hozo et al. [11]

### 2.5. Statistical Analysis

Meta-analysis was performed using the soft-ware package RevMan5.2. Statistical calculations were performed for all eligible studies with detailed data of the LIPUS and control groups. The descriptive statistics, one sample, or independent samples of comparing means were used for the analysis, and the results were reported as mean ± SD. Continuous variables were presented as mean values. Begg's test was performed to detect publication bias [12]. Statistical heterogeneity was assessed using the I^2^ [13]. Low heterogeneity was considered when I^2^ was ranged from 25% to 50%; moderate heterogeneity when I^2^ ranged from 50% to 75%; and high degree of heterogeneity when I^2^ over 75%. In cases of I^2^ larger than 50%, a random-effect model was used. Otherwise, a fixed-effect model was used. Significance was set at P < 0.05.

## 3. Results

### 3.1. Study Selection

**Figure 1:**
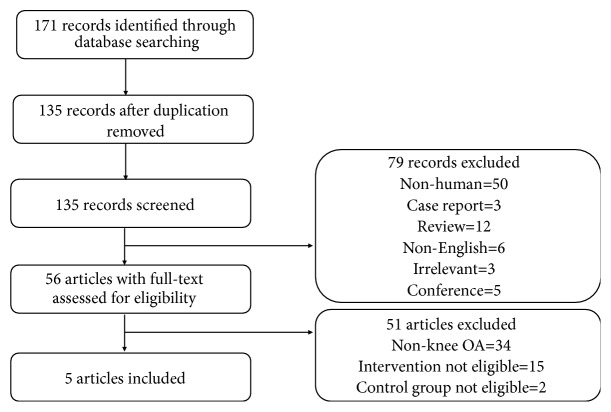
Flowchart for the selection of included trials.

Overall, 171 potentially records were identified through database searching and 135 abstracts were remained after removing duplication recordings. Then, abstracts were reviewed for preliminary assessment, after which 56 full-text articles were evaluated for eligibility. Of the 56 articles, 5 met the inclusion criteria and other 51 article were excluded. Among these 51 articles, 15 articles were not eligible for the intervention, 34 articles were treating patients not with knee OA, and 2 of them were not eligible for control group (Figure 1).

### 3.2. Characteristics of Studies

The 5 selected studies involved totally 288 participants (143 in control group and 145 in LIPUS group, separately) [14–18]. The basic characteristics of these trials were demonstrated in Table 1. The mean age of the participants was approximately 60 years old. Mean body mass index (BMI) in 4 of the trails was about 30 (except BMI in the study carried out by Jia et al. was around 25). The study by Loyola et al. used Osteoarthritis Research Society International (OARSI) atlas classification grade to report Joint Space Narrowing. The other 4 trials applied Kellgren-Lawrence Grade to describe radiological severity of knee OA and all the participants belonged to Grades II-III. Treatment duration in 4 of the trials lasted 10 days, and only the study carried out by Loyola et al. used LIPUS treatment for 8 weeks. Methodological qualities were assessed with Jadad scale. Of all the 5 included studies, 4 reported randomization, double-blind, and withdraw. Only the study carried out by Yildiz et al. did not refer to the loss of follow-up (Table 2). No significant publication bias was found in the selected studies.

### 3.3. Functional Recovery

**Figure 2:**
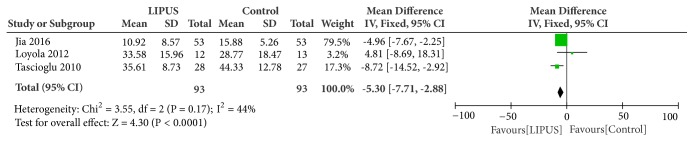
Forest plot demonstrated the effect of LIPUS on functional recovery evaluated by WOMAC scale.

Three of the selected studies evaluated the functional recovery in patients with knee OA using WOMAC scale. The data demonstrated a beneficial effect of LIPUS on improvement of the function of knee. Pooling of the data about WOMAC score in these trails showed a mean reduction of 5.30 points (95%CI, 2.88 to 7.71 points; I^2^ = 44%, heterogeneity: P = 0.17) in measuring knee function, indicating evidence with moderate quality on the benefit effect of LIPUS on functional recovery in patients with knee OA (Figure 2).

**Figure 3:**
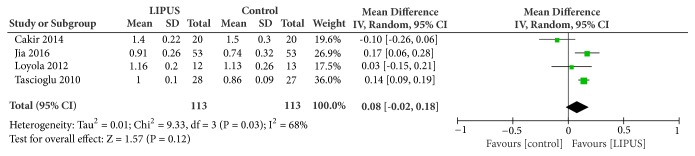
Forest plot demonstrated the effect of LIPUS on functional recovery evaluated by ambulation speed.

Four of the studies measured AS or 20/50 meter walking time to assess functional recovery. After being transformed into speed, the result of pooling demonstrated no significant improvement in walking speed. The mean walking speed was increased by 0.08 m/s under LIPUS stimulation (95% CI, -0.02 to 0.18 m/s; I^2^ = 68%, heterogeneity: P = 0.03) (Figure 3).

### 3.4. Pain Intensity

**Figure 4:**
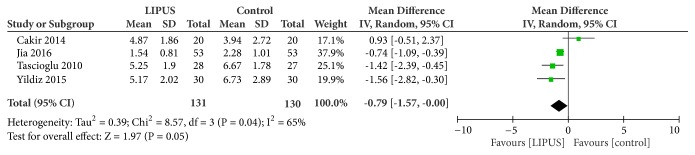
Forest plot demonstrated the effect of LIPUS on pain relief evaluated by VAS.

Four trials assessed the effect of LIPUS on pain relief in patients with knee OA. One study by Cakir et al. did not demonstrate beneficial effect of LIPUS on pain relief. Pooling of the scores of VAS revealed a mean decrease of 0.79 points (95% CI, -1.57 to 0.00 points; I^2^ = 65%, heterogeneity: P = 0.04) as a result of LIPUS treatment on patients with knee OA with moderate heterogeneity (Figure 4).

## 4. Discussion

The present meta-analysis focused on the effects of LIPUS in patients with knee OA. Five trials which specifically described the effectiveness of LIPUS on knee OA were included after we searched relevant RCT studies.

Pain management was a great concern for doctors in treating knee OA [19, 20]. VAS scale was most reported to evaluate pain intensity (4 trials, n = 261) in the selected studies. Pooling showed a mean reduction of 0.78 points under LIPUS stimulation without any known adverse event, indicating that LIPUS had a beneficial effect on pain relief and a promising safety in treating patients with knee OA. The study by Jia et al. combined LIPUS with NSAIDs (diclofenac) to treat knee OA, while the other studies did not administrate drugs as an intervention. Thus, the VAS score in the study by Jia et al. at the endpoint was apparently lower than the other 3 studies which explained the highest weight (79%) in the 4 studies. Costa et al. reported that NSAID therapy such as diclofenac had a moderate to large effect on pain intensity according to the results of VAS scale [21]. This information suggested that LIPUS could be used as an effective adjuvant method with/without other therapies such as NSAID to manage pain intensity. On the other hand, the study by Cakir et al. combined LIPUS treatment with home exercise training, which might be one reason for the source of heterogeneity.

Functional recovery was another primary outcome in these studies to identify the improvement in treating knee OA by LIPUS. Overall, LIPUS presented a positive effect on knee function evaluated by WOMAC scale. One of the studies by Loyola et al. did not demonstrate significant benefit on functional improvement by LIPUS. However, the baseline of his study showed that the mean WOMAC score was 31.42 ± 19.67 in control group and 40.09 ± 10.37 in LIPUS group, which could explain the source of the heterogeneity. Alternatively, many researches have reported that LIPUS could induce extracellular matrix synthesis and increase rates of chondrocyte migration and proliferation [22–24], supporting that LIPUS has a chondroprotective effect in cellular experiments. In addition, data from animal experiments also showed that LIPUS could increase the synthesis of type II collagen in articular cartilage and exhibited the ability to attenuate the progression of cartilage degeneration in OA in different animal models. The intervention of LIPUS was suggested as early as possible by Gurkan et al. [25, 26].

A trend in favor of LIPUS without significant difference was found in AS after pooling the 4 trials. The study by Cakir et al. and Loyola et al. reported that LIPUS had no significant effect on the improvement of walking speed. The possible reason might be that the number of patients enrolled in these 2 studies is not enough to make a clear conclusion (23 in Loyola et al. and 40 in Cakir et al.). As contrast, 106 and 55 patients were enrolled by Jia and Tascioglu's study, separately. In addition, the participants in Cakir's study were asked to perform home exercise at least 3 times a week. Ulus et al. found that the use of ultrasound combined with conventional physical therapy programs had no further significant effect in patients with knee OA and they concluded that the effect of ultrasound could be masked by exercises [27]. On the other hand, Rutjes et al. reported that therapeutic ultrasound might be beneficial on pain relief and functional recovery for patients with knee OA [28]. We interpret these results to mean that sufficient number of the participants and adequate intervention are needed to export a stable outcome.

The present study has several limitations. Firstly, the parameters of LIPUS device in the selected 5 trials were varied from 120 mW/cm^2^ to 500 mW/cm^2^, and the endpoint of observation differed from 10 days to 8 weeks, which might contribute to the existence of heterogeneity. Secondly, several studies measured walking ability by different methods such as 50-meter walking time, 20-meter walking time, and 6-minute walking distance. Different walking distance during measurement might result in heterogeneity when pooling together to analyze. Thirdly, the present study only focused on pain relief and functional recovery; other indices such as the changes of radiological parameters should be further investigated.

## 5. Conclusion

The present study includes 5 high quality randomized controlled trials. The result indicated that LIPUS, used to treat knee OA without any adverse effect, had a beneficial effect on pain relief and knee functional recovery. More evidence is needed to prove whether LIPUS is effective in improving walking ability.

## Figures and Tables

**Table 1:** Characteristics of the selected studies.

**Author**	**Year**	**N** **(Control/LIPUS)**	**Age**		**BMI**		**Kellgren-Lawrence Grade (I/II/III/IV)**		**Interested outcome**	**Treatment duration**	**JADAD**
			**Control**	**LIPUS**	**Control**	**LIPUS**	**Control**	**LIPUS**			
Jia et al.	2016	53/53	61.3 ± 10.2	63.4 ± 9.7	26.2 ± 5.9	25.8 ± 3.4	0/44/9/0	0/46/7/0	VAS, LI, ROM WOMAC, 50 meter walking time	10 days	5

Loyola et al.	2012	13/14	61.1 ± 11.5	62.5 ± 9.5	30.4 ± 5.7	34.0 ± 8.5	/	/	WOMAC, 6 Min Walk	8 weeks	5

Cakir et al.	2014	20/20	58.2 ± 9.9	57.1 ± 7.8	30.9 ± 4.0	29.5 ± 5.9	All II-III	All II-III	VAS, WOMAC, 20m walking time	10 days	5

Yildiz et al	2015	30/30	57.7 ± 7.1	54.6 ± 6.5	30.9 ± 4.3	31.1 ± 4.7	0/16/14/0	0/10/20/0	VAS, LI, ROM	10 days	4

Tascioglu et al.	2010	27/28	60.0 ± 2.8	61.6 ± 3.7	28.7 ± 4.0	30.8 ± 3.8	0/15/12/0	0/16/12/0	VAS, WOMAC, 20m walking time	10 days	5

**Table 2:** Specific item of JADAD scale for selected studies.

**Author**	**Year**	**N** **(Control/LIPUS)**	**Random methods**	**Blind**	**Lost**	**Total**
Jia et al.	2016	53/53	2	2	1	5
Loyola et al.	2012	13/14	2	2	1	5
Cakir et al.	2014	20/20	2	2	1	5
Yildiz et al	2015	30/30	2	2	0	4
Tascioglu et al.	2010	27/28	2	2	1	5

## Appendix

### A. Search Strategies for Pubmed, Embase and Cochrane Library

#### A.1. Pubmed

  #1 osteoarthro∗[tiab] or gonarthriti∗[tiab] or gonarthro∗[tiab] or osteo?arthritis[tiab]
  #2 (knee∗[tiab] or joint∗[tiab]) and (pain∗[tiab] or discomfort∗[tiab])
  #3 (knee∗[tiab] or joint∗[tiab]) and stiff∗[tiab]
  #4 #1 or #2 or #3
  #5 pulsed ultrasound[tiab]
  #6 LIPUS[tiab]
  #7 #5 or #6
  #8 #4 AND #7

#### A.2. Embase

  #1 'osteoarthriti$':ab,ti OR 'osteoarthro$':ab,ti OR 'osteo?arthritic':ab,ti OR 'gonarthriti$':ab,ti OR 'gonarthro$':ab,ti OR 'joint$) adj3 (pain$':ab,ti OR 'ach$':ab,ti OR 'discomfort$))':ab,ti OR '((knee$':ab,ti OR 'joint$) adj3 stiff$)':ab,ti
  #2 'pulsed ultrasound':ab,ti OR 'lipus':ab,ti
  #3 #1 and #2

#### A.3. Cochrane Library

  #1 (osteoarthritis∗ or osteoarthro∗ or gonarthriti∗ or gonarthro∗ or arthros∗ or arthrot∗ or ((knee∗ or joint∗) near/3 (pain∗ or ach∗ or discomfort∗)) or ((knee∗OR joint∗) near/3 stiff∗))
  #2 pulsed ultrasound
  #3 LIPUS
  #4 #2 or #3
  #5 #1 and #4

## Abbreviations

OARSI: Osteoarthritis Research Society International
BMI: Body mass index
AS: Ambulation speed
LIPUS: Low-intensity pulsed ultrasound
OA: Osteoarthritis.
